# Supplementary material for: High speciation in the cryptic Pristimantis celator clade (Anura: Strabomantidae) of the Mira river basin, Ecuador-Colombia
Source: PeerJ. 2025 Jan 29;13:e18680. doi: 10.7717/peerj.18680 (PMC11786716; doi:10.7717/peerj.18680)
Supplement: Supplemental Information 1 [file peerj-13-18680-s001.docx]

**Supplementary Material 2. Redescription of *Pristimantis verecundus***

***Pristimantis verecundus* (Lynch & Burrowes, 1990)**

*Eleutherodactylus verecundus* Lynch & Burrowes, 1990, Occas. Pap. Mus. Nat. Hist. Univ. Kansas, 136: 26. Holotype: IND-AN 1834, by original designation. Type locality: "Reserva La Planada, Mcipio. Ricuarte, Depto. Nariño, Colombia, 1780 m".

*Eleutherodactylus* (*Eleutherodactylus*) *verecundus —* Lynch & Duellman, 1997, Univ. Kansas Mus. Nat. Hist. Spec. Publ., 23: 234.

*Pristimantis verecundus —* Heinicke, Duellman, & Hedges, 2007, Proc. Natl. Acad. Sci. USA, Suppl. Inform., 104: Table 2.

*Pristimantis* (*Pristimantis*) *verecundus —* Hedges, Duellman, and Heinicke, 2008, Zootaxa, 1737: *128.*

*Pristimantis* (*Trachyphrynus*) *verecundus —* Yánez-Muñoz, Reyes-Puig, Reyes-Puig, Lagla-Chimba, Paucar-Veintimilla, Urgiles-Merchán y Carrión-Olmedo, 2024, This work.

***Common name in Spanish:*** *Cutín de Zacualtipan*

***Suggested common English name:*** *Zacualtipan Robber Frog*

**Holotype (**Fig. supp. 1**).** IAvH-AM-1834, an adult female, collected at Reserva Natural La Planada, Municipio de Ricaurte, Departamento Nariño, Colombia, 1780 m, on 26 April 1986 by Patricia. A. Burrowes.

**Revised paratype (**Fig. supp. 1**).** adult female, IAvH-AM-1457.

**Material examined (**Fig. supp. 1**).** Nine specimens, four females, three males and two juveniles referred to in Appendix 1.

**Diagnosis:** *Pristimantis verecundus* is a member of the *Pristimantis myersi* species group and *P. celator* species clade, characterized by the following combination of characters: (1) Skin of dorsum shagreen with low and scattered flat warts, venter areolate; discoidal fold present; dorsolateral glandular folds present, short, only reaching the sacrum, at the anterior border of ilium; flanks and upper surfaces of limbs bear low tubercles; transverse glandular tibial stripes; (2); tympanic annulus visible, round, length 35.25% length of eye (n= 9); (3) snout rounded in dorsal and lateral view;(4) upper eyelid bearing one enlarged subconic to conical tubercle, narrower than IOD; cranial crests absent; (5) vomerine odontophores present, oblique in outline; (6) males having vocal slits and vocal sac; nuptial pads absent; (7) first finger shorter than second; discs broad 2X the width of the digits; (8) fingers bearing lateral fringes (9) two large ulnar tubercles; (10) heel bearing conical tubercles; inner edge of tarsus bearing short fold; (11) inner metatarsal fold present, short, inner metatarsal tubercle oval, 2X the size of the rounded external one; supernumerary palmar tubercles low; (12) toes bearing lateral fringes; webbing absent; fifth toe longer than third; discs expanded, 2X the width of the digits (13) dorsum in brown to dark brown with yellowish brown to brick red dorsolateral folds; venter brown with cream or light brown flecks and with a distinctive V-shaped mark on the throat; (14) SVL males 17.56-19.24 mm (mean = 19.05, n= 3), females SVL= 18.5-22.84 mm (mean = 20.28 mm, n= 6)

**Comparisons with other species (**Figs. 2, 6, 8**).** *Pristimantis verecundus* is easily distinguishable from more closely related species (*P. robayoi* sp. nov. *P. mutabilis*, *P. satheri* sp. nov., *P. broaddus* and *P. praemortuus*) by the following characters. Digital pads of toes expanded, wider than the digit, 2X or more the width of the digit in *P. verecundus* (digital pads of toes narrow, about the width of the digit in *P. broaddus* sp. nov. and *P. praemortuus* sp. nov.). Upper eyelid bearing one enlarged subconic to conical tubercle in *P. verecundus* (several small, rounded tubercles in *P. satheri* sp. nov.). Dorsolateral folds short, only reaching the sacrum, at the anterior border of ilium in *P. verecundus* (extending posteriorly beyond the sacrum in *P. mutabilis* and extending to the sacrum, with two distinct black subconical scapular tubercles in *P. robayoi* sp. nov.). Distinctive V-shaped throat marking in *P. verecundus* (absent in *P. mutabilis*, *P. robayoi* sp. nov. and *P. satheri* sp. nov.).

**Description of the holotype (**Fig. supp. 1, 2, 6**).** Adult female. Measurements the same as the original description. Head slightly longer than wide (9.00 mm vs 9.3 mm); head width 40% of SVL; head length 41.3% of SVL; snout rounded in dorsal and lateral view; eye–nostril distance 11.1% of SVL; canthus rostralis concave in lateral view, nostrils directed laterally; interorbital slightly convex, narrower than the upper eyelid; cranial crests absent; upper eyelid with one conspicuous conical tubercle on the center of the eyelid (reduced by preservation effects); upper eyelid width 87.5% of IOD; tympanic membrane and tympanic annulus differentiated, present, supratympanic fold present, which covers less than 10% of the tympanic annulus; tympanum diameter 33.3% of eye diameter; with one conical postrictal tubercle, with other lower subconical. Choanae moderate in size, not concealed by palatal shelf of maxilla; dentigerous processes of vomer present, subtriangular in outline, moderately separated, with three to four teeth; tongue longer than wide.

Skin on dorsum shagreen, bearing low and scattered warts; dorsolateral fold present, glandular, short, extending from the scapular region and only reaching the sacrum, at the anterior border of ilium; skin on venter areolate; discoidal fold present; cloaca with low warts. Forearms slender, subconical ulnar tubercles present; palmar tubercle bilobed, 1.5X the size of the thenar which is oval; subarticular tubercles rounded, defined; supernumerary tubercles indistinguishable; fingers with lateral fringes; Finger I shorter than Finger II; disc on Finger I slightly wider than the digit, discs expanded, more than twice the width of the digits in Fingers II to IV, circumferential grooves present.

Hindlimbs slender, tibia length 62% of SVL; foot length 49.7% of SVL; upper surfaces of hindlimbs shagreen, with transversal rows of glandular tissue on the tibia; ventral surfaces areolate, heel bearing one conical tubercle, conspicuous; inner tarsal fold present; inner oval, 2.5X times the outer metatarsal tubercle that is rounded; supernumerary plantar tubercles slightly visible; toes with lateral fringes, narrow; interdigital membrane absent basal; discs of digits expanded; two times the width of the digits on toes I to V; all toes circumferential grooves; disc on Toe V do not exceed the distal subarticular tubercle on Toe IV

**Color of holotype in ethanol 70% (Fig. sup. 1):** The dorsum is drab-gray (256) mottled with darker burn umber (49) marks scattered across the body. Dorsal surface of the limbs is drab-gray (256) with faint darker markings, forming transverse irregular pattern. Dorsolateral folds, transverse rows of glandular surfaces on the tibia and dorsal surfaces of toes cream. Venter and throat mikado brown (42) marbled with raw umber (280), throat with a distinctive “V”-shaped raw umber (23), delimited with two mikado brown (42) diagonal stripes. Underbelly and groin with cream patches. Iris dark grey.

**Variation (**Fig. supp. 3, 6, 7**)**. *Pristimantis verecundus* sp. nov. shows sexual dimorphism in body size and coloration (Fig. supp.3). Males of this species between 1.5–1.18 times smaller in SVL than females (Fig. supp.3). Other morphometric variation is showing in the Table 2. In life (Fig. supp. 7), variation can be examined in Supplementary Material Fig.Sup7. Dorsum drab-gray (256) (IAvH1834), fawn color (258) (IAvH1457) to dark gray (299) with white dots (DHMECN 19433, DHMECN 5188), with burn umber (49) dorsal irregular marks. With cream dorsolateral folds (I, IAvH1457, DHMECN 5188), to dark gray (299) with white dots (DHMECN 19433). Diagonal subocular bands from burnt umber (49) (IAvH1834, IAvH1457) to jet black (300) with white dots (DHMECN 19433, DHMECN 5188). Belly mikado brown (42) (IAvH1834, IAvH1457), sepia (279) (DHMECN 19433), and olive brown (278) (DHMECN 5188), marbled with raw umber (280) throughout belly. Throat with distinctive V-shaped marking from raw umber (23) (IAvH1834, IAvH1457) to sepia (279) (DHMECN 19433, DHMECN 5188). Groin cream (IAvH1834, IAvH1457) to mikado brown (42) (DHMECN 5188). In preservative (Fig. supp. 6), variation can be examined in Supplementary Material Fig.Sup6. Dorsum from cinnamon-rufous (31) (DHMECN 17891), burnt sienna (38) (DHMECN 12599, 12500, 12597, 12601), maroon (39) (DHMECN 17892) to sepia (279) (DHMECN 16569). With dorsolateral glandular folds from robin rufous (29) (DHMECN 12599), burnt orange (10) (DHMECN 17891, DHMECN 15007, DHMECN 12500, DHMECN 12597, DHMECN 12601), to creamy yellow (82) (DHMECN 16569). With pigmented canthal bands of the same color as the dorsolateral folds in some specimens (DHMECN 12500, DHMECN 16569). Diagonal subocular bands from maroon (39) (DHMECN 12599, DHMECN 17892, DHMECN 17891, DHMECN 12500, DHMECN 16569, DHMECN 12597, DHMECN 12601) to jet black (300) (DHMECN 15007). Digits of the same color as dorsolateral folds, and dorsally brown digital discs. Belly pale buff (1) (DHMECN 12599), mikado brown (42) (DHMECN 12597), creamy yellow (82) (DHMECN 15007), with thick burnt sienna (38) marbled marks throughout belly. Throat with a distinctive burnt sienna V-shaped mark.

**Distribution and natural history (**Fig. 2**).** *Pristimantis verecundus* is known to inhabit western low montane forests from the Güiza River Basin in southwestern Colombia to the San Juan River Basin in northwestern Ecuador. In Colombia, all documented occurrences are situated at elevations between 1,700 and 1,930 meters, within the Mira river basin. This species is found in the low montane evergreen forest of the Western Cordillera of the Andes (MAE 2013), which is characterized by a closed canopy with trees reaching up to 20 meters in height. The forest is covered by a diverse array of epiphytes, orchids, bromeliads, bryophytes, and ferns. This species is active at night, perching on the leaves of shrubs in the lower and middle stratum of the forest, at elevations between 100 and 160 cm. It was found in sympatry with four other species: the *Pristimantis pteridophilus* complex, *P. apiculatus*, *P. hectus*, and *P. robayoi* sp. nov. (Fig. 3).

**Remarks.** The original description of *Pristimantis verecundus* (Lynch & Burrowes, 1990) is based on the entire type series and therefore includes morphological features of *P. satheri* sp. nov. (IAvH-Am-1492) and *P. robayoi* sp. nov. (IAvH-1801) and not only the holotype specimen (IAvH-Am 1834). Due to the uncertainty of the material referred to in the description, we cannot recognize which features correspond to the holotype. Furthermore, the photograph presented in Figure 3 of the original description corresponds to *P. robayoi* sp. nov. (IAvH-1801). The description of the coloration in life does not detail on which specimens it was based and we note that no mention is made of the presence of reddish groins, however, photographs of the type of material of *P. verecundus* (IAvH-Am 1457, IAvH-Am 1834) show groin markings that could potentially have been reddish. As the etymology of the species indicates, the epithet *verecundus* refers to the fact that the authors found no distinctive coloration for the species. Our observations of fresh material have allowed us to identify the presence of red groins as a variable character, present in some specimens and not in others.
