# Supplementary material for: High speciation in the cryptic Pristimantis celator clade (Anura: Strabomantidae) of the Mira river basin, Ecuador-Colombia
Source: PeerJ. 2025 Jan 29;13:e18680. doi: 10.7717/peerj.18680 (PMC11786716; doi:10.7717/peerj.18680)
Supplement: Supplemental Information 2 [file peerj-13-18680-s002.docx]

Supplemental Table 1. GenBank accession number of the sequences generated in this study.

| **Species** | **Citation** | **Type of Type** | **Specimen Catalog** | **Genbank Accession Number** | **GenSeq Nomenclature** |
| --- | --- | --- | --- | --- | --- |
| *Pristimantis praemortua* sp. nov. | This paper | Paratype | DHMECN19557 | PQ189053 | genseq-2 |
|  |  | Paratype | DHMECN19545 | PQ189054 | genseq-2 |
|  |  | Paratype | DHMECN19577 | PQ189055 | genseq-2 |
|  |  | Paratype | DHMECN19579 | PQ189056 | genseq-2 |
| *Pristimantis broaddus* sp. nov. | This paper | Holotype | DHMECN19037 | PQ189057 | genseq-1 |
|  |  | Paratype | DHMECN19028 | PQ189058 | genseq-2 |
|  |  | Paratype | DHMECN19029 | PQ189059 | genseq-2 |
|  |  | Paratype | DHMECN19031 | PQ189060 | genseq-2 |
|  |  | Paratype | DHMECN19032 | PQ189061 | genseq-2 |
|  |  | Paratype | DHMECN19033 | PQ189062 | genseq-2 |
|  |  | Paratype | DHMECN19034 | PQ189063 | genseq-2 |
|  |  | Paratype | DHMECN19035 | PQ189064 | genseq-2 |
|  |  | Paratype | DHMECN19434 | PQ189065 | genseq-2 |
| *Pristimantis robayoi* sp. nov. | This paper | Holotype | DHMECN17894 | PQ189066 | genseq-1 |
|  |  | Paratype | DHMECN16567 | PQ189067 | genseq-2 |
|  |  | Paratype | DHMECN16573 | PQ189068 | genseq-2 |
|  |  | Paratype | DHMECN16575 | PQ189069 | genseq-2 |
|  |  | Paratype | DHMECN19429 | PQ189070 | genseq-2 |
| *Pristimantis verecundus* | This paper | Non-Type | DHMECN16568 | PQ189071 | genseq-3 |
|  |  | Non-Type | DHMECN16569 | PQ189072 | genseq-3 |
|  |  | Non-Type | DHMECN17891 | PQ189073 | genseq-3 |
|  |  | Non-Type | DHMECN17892 | PQ189074 | genseq-3 |
|  |  | Non-Type | DHMECN19430 | PQ189075 | genseq-3 |
|  |  | Non-Type | DHMECN19431 | PQ189076 | genseq-3 |
|  |  | Non-Type | DHMECN19432 | PQ189077 | genseq-3 |
|  |  | Non-Type | DHMECN19433 | PQ189078 | genseq-3 |
| *Pristimantis* csp. 2 | This paper | Non-Type | DHMECN16570 | PQ189079 | genseq-3 |
|  |  | Non-Type | DHMECN16576 | PQ189080 | genseq-3 |
| *Pristimantis* csp. 4 | This paper | Non-Type | DHMECN16572 | PQ189081 | genseq-3 |
