# Supplementary material for: High speciation in the cryptic Pristimantis celator clade (Anura: Strabomantidae) of the Mira river basin, Ecuador-Colombia
Source: PeerJ. 2025 Jan 29;13:e18680. doi: 10.7717/peerj.18680 (PMC11786716; doi:10.7717/peerj.18680)
Supplement: Supplemental Information 3 [file peerj-13-18680-s003.pdf]

	Pristimantis sp 1	*Pristimantis macarthur sp. nov.*	*Pristimantis broaddus sp. nov.*	*Pristimantis satheri sp. nov.*	*Pristimantis sp 2*	*Pristimantis celator*	*Pristimantis mutabilis*	*Pristimantis sp 3*	*Pristimantis sp 4*	*Pristimantis sp 5*	*Pristimantis sp 6*	*Pristimantis sp 7*	*Pristimantis robayoi sp. nov.*
Pristimantis sp 1													
Pristimantis macarthur sp. nov.	6.33%												
Pristimantis broaddus sp. nov.	7.19%	3.54%											
Pristimantis satheri sp. nov.	11.17%	10.01%	11.00%										
Pristimantis sp 2	12.50%	12.38%	12.54%	11.14%									
Pristimantis celator	12.14%	11.46%	12.47%	12.11%	14.20%								
Pristimantis mutabilis	12.46%	11.67%	12.28%	11.14%	15.12%	11.06%							
Pristimantis sp 3	11.71%	12.28%	12.74%	13.04%	15.04%	10.83%	10.05%						
Pristimantis sp 4	12.50%	12.49%	12.81%	12.99%	14.97%	10.49%	9.56%	6.12%					
Pristimantis sp 5	15.06%	12.40%	13.77%	14.05%	15.63%	11.63%	9.87%	6.70%	5.36%				
Pristimantis sp 6	12.16%	12.08%	12.74%	13.26%	14.79%	10.79%	10.89%	4.84%	5.83%	8.53%			
Pristimantis sp 7	11.44%	11.60%	12.17%	12.80%	15.23%	9.81%	9.65%	4.18%	5.24%	5.58%	3.09%		
Pristimantis robayoi sp. nov.	13.21%	11.61%	11.87%	12.59%	14.00%	11.20%	11.00%	9.28%	8.79%	9.61%	8.08%	7.79%	
Pristimantis verecundus	11.72%	10.97%	11.42%	12.58%	14.79%	9.56%	10.14%	9.30%	9.31%	9.59%	9.48%	8.08%	8.81%

S2. Estimates of Evolutionary Divergence over Sequence Pairs between Groups. The number of base differences per site from averaging over all sequence pairs between groups are shown. This analysis involved 60 nucleotide sequences. All ambiguous positions were removed for each sequence pair (pairwise deletion option). There were a total of 1538 positions in the final dataset. Evolutionary analyses were conducted in MEGA11
