# Supplementary figures and images for: High speciation in the cryptic Pristimantis celator clade (Anura: Strabomantidae) of the Mira river basin, Ecuador-Colombia
Source: PeerJ. 2025 Jan 29;13:e18680. doi: 10.7717/peerj.18680 (PMC11786716; doi:10.7717/peerj.18680)

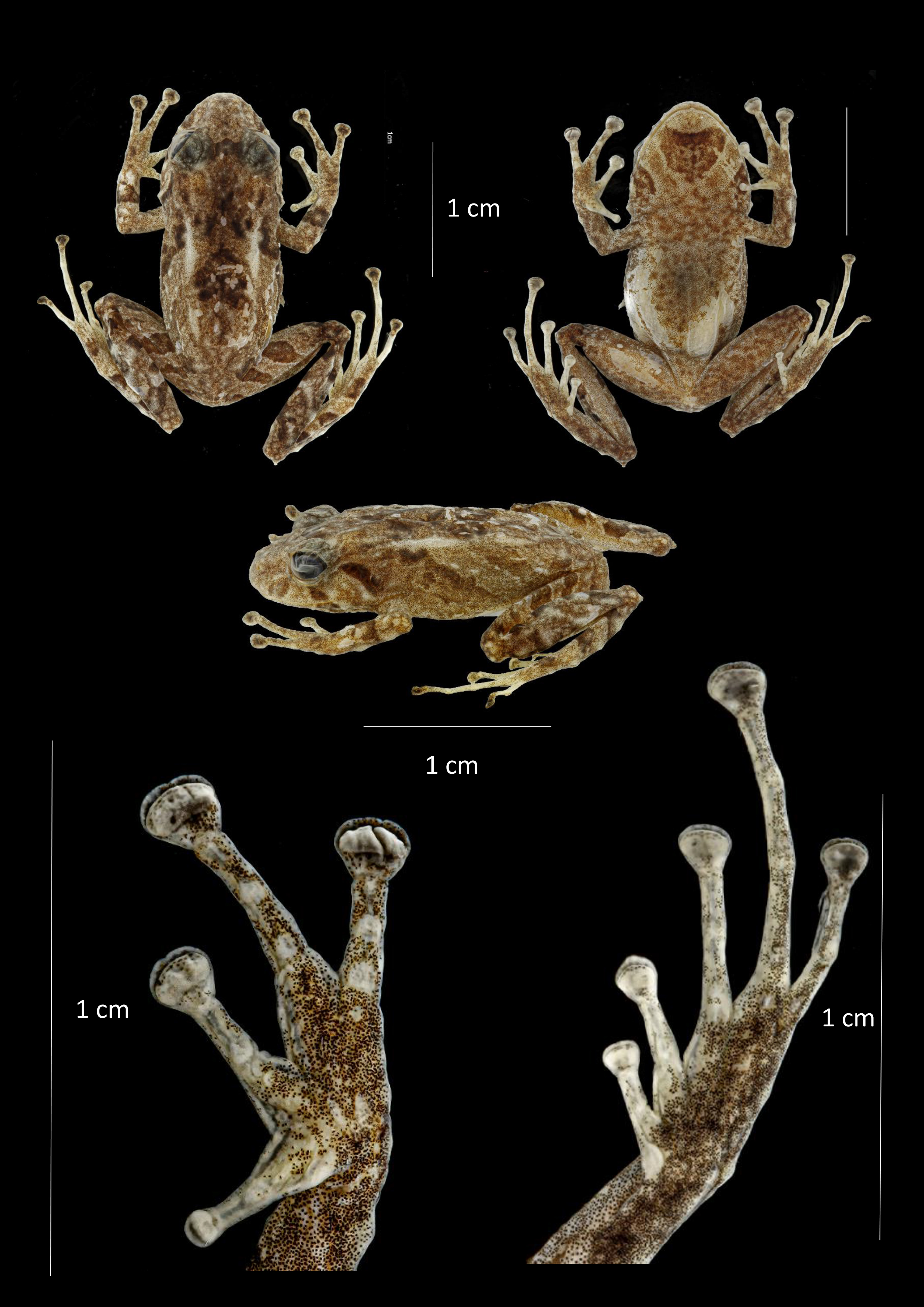

Supplement: Supplemental Information 4 — Photographs by Kristian Venegas-Valencia and Sandra P. Galeano. [file peerj-13-18680-s004.jpg]

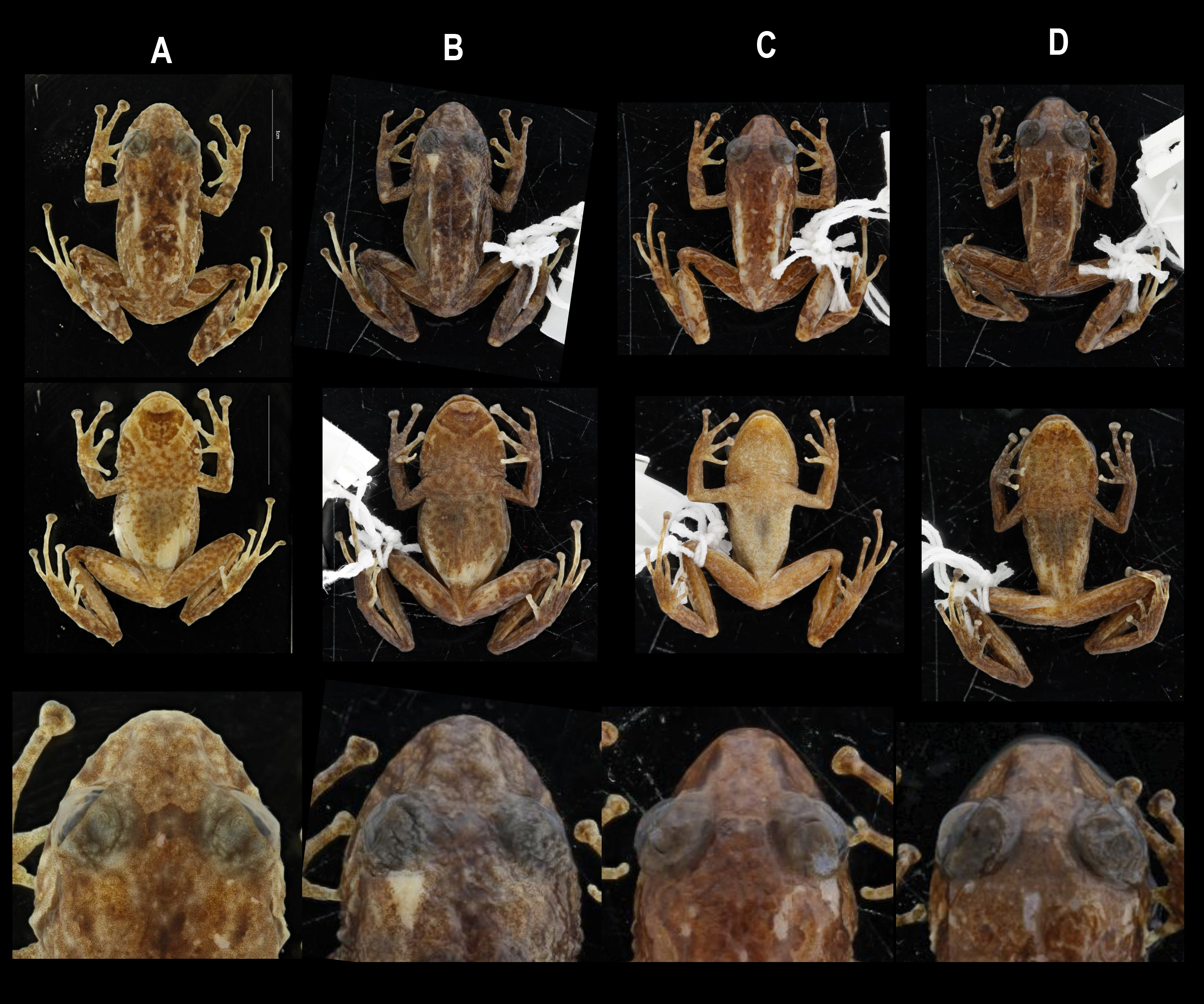

Supplement: Supplemental Information 5 — Holotype (A) IAvH 1801; (B) Paratype IAvH1457; (C) new allocation P. sathreri sp. nov., IAvH-Am-1492; (D) new allocation Pristimantis robayoi sp. nov., IAvH1801. Photographs by Kristian Venegas-Valencia and Sandra P. Galeano. [file peerj-13-18680-s005.jpg]

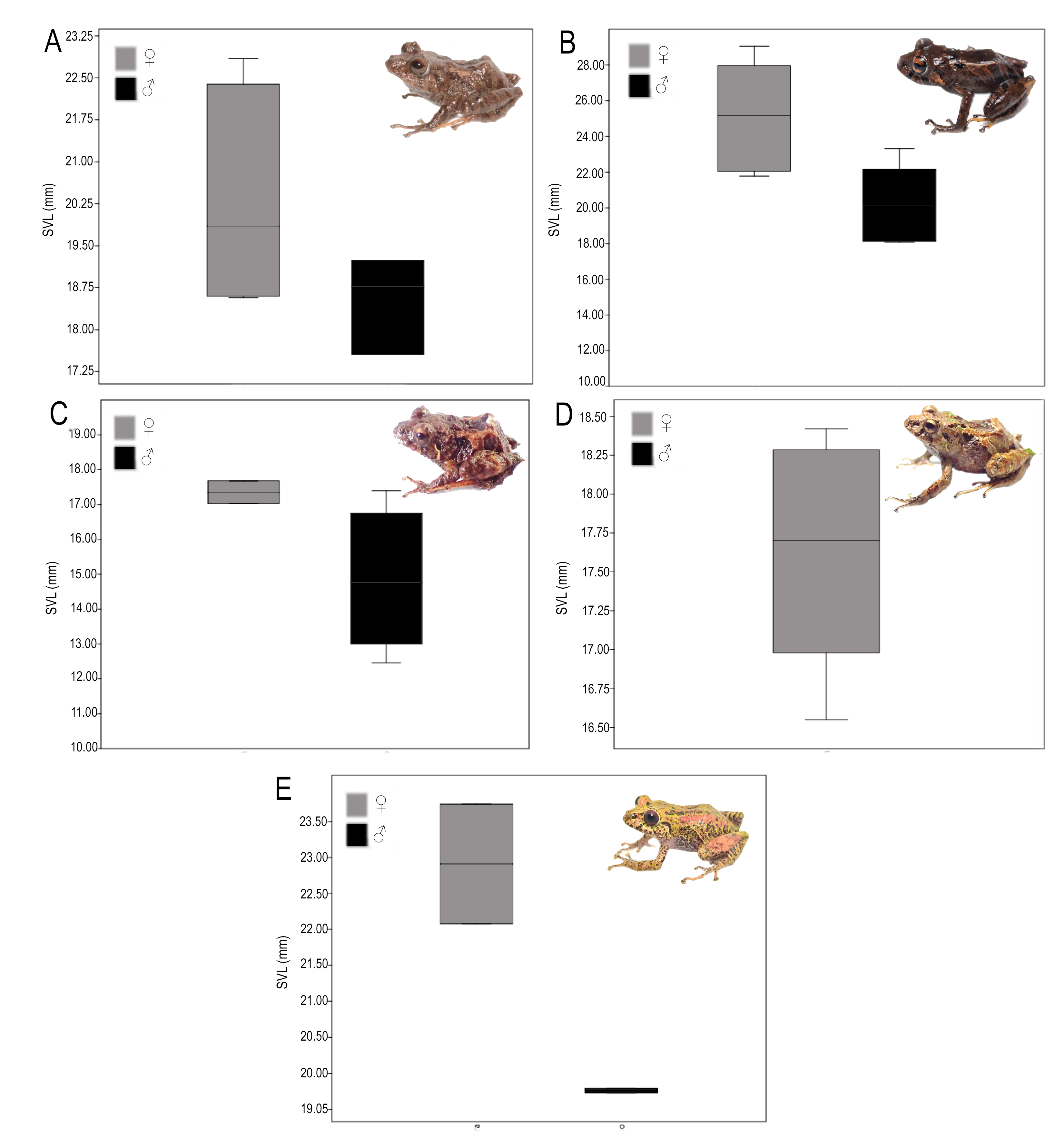

Supplement: Supplemental Information 6 — (A) P. verecundus; (B) P. robayoi sp. nov. (C) P. praemortuus sp. nov.; (D) P. broaddus sp. nov.; (E) P. satheri [i]sp. nov. Illustration by Mario H. Yánez-Muñoz. [file peerj-13-18680-s006.jpg]

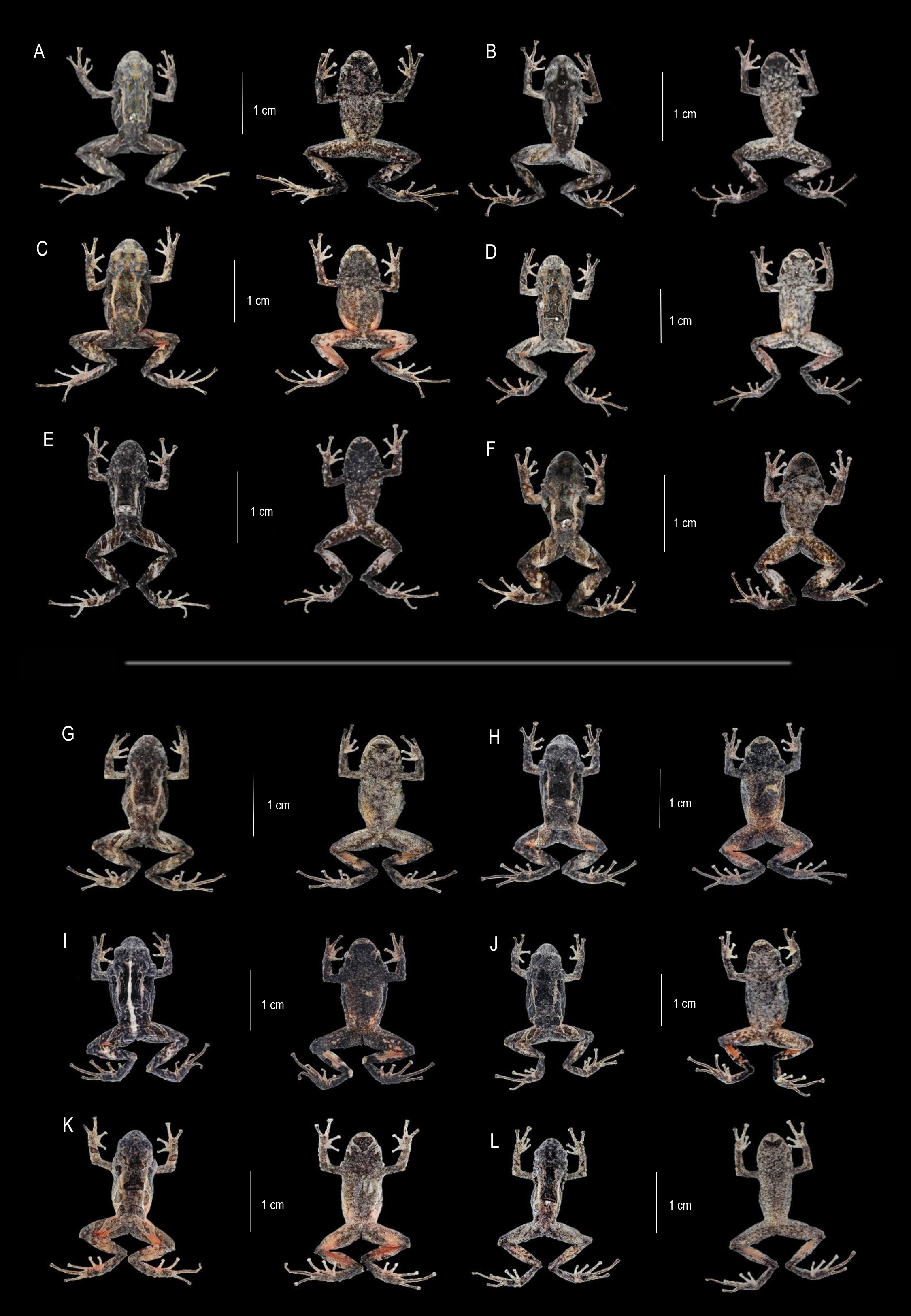

Supplement: Supplemental Information 7 — Holotype: (A) DHMECN 19591. Paratypes: (B) DHMECN 19535, (C) DHMECN 19557, (D) DHMECN 19570, (E) DHMECN 19547, (F) DHMECN 19546; Variation of type series of Pristimantis boraddus sp. nov. Holotype: (G) DHMECN 19037; Paratype: DHMECN: (H) DHMECN 19031, (I) DHMECN 19036, (J) DHMECN 19032, (K) DHMECN 19035, (L) DHMECN 19028. Photographs by Christian Paucar. [file peerj-13-18680-s007.jpg]

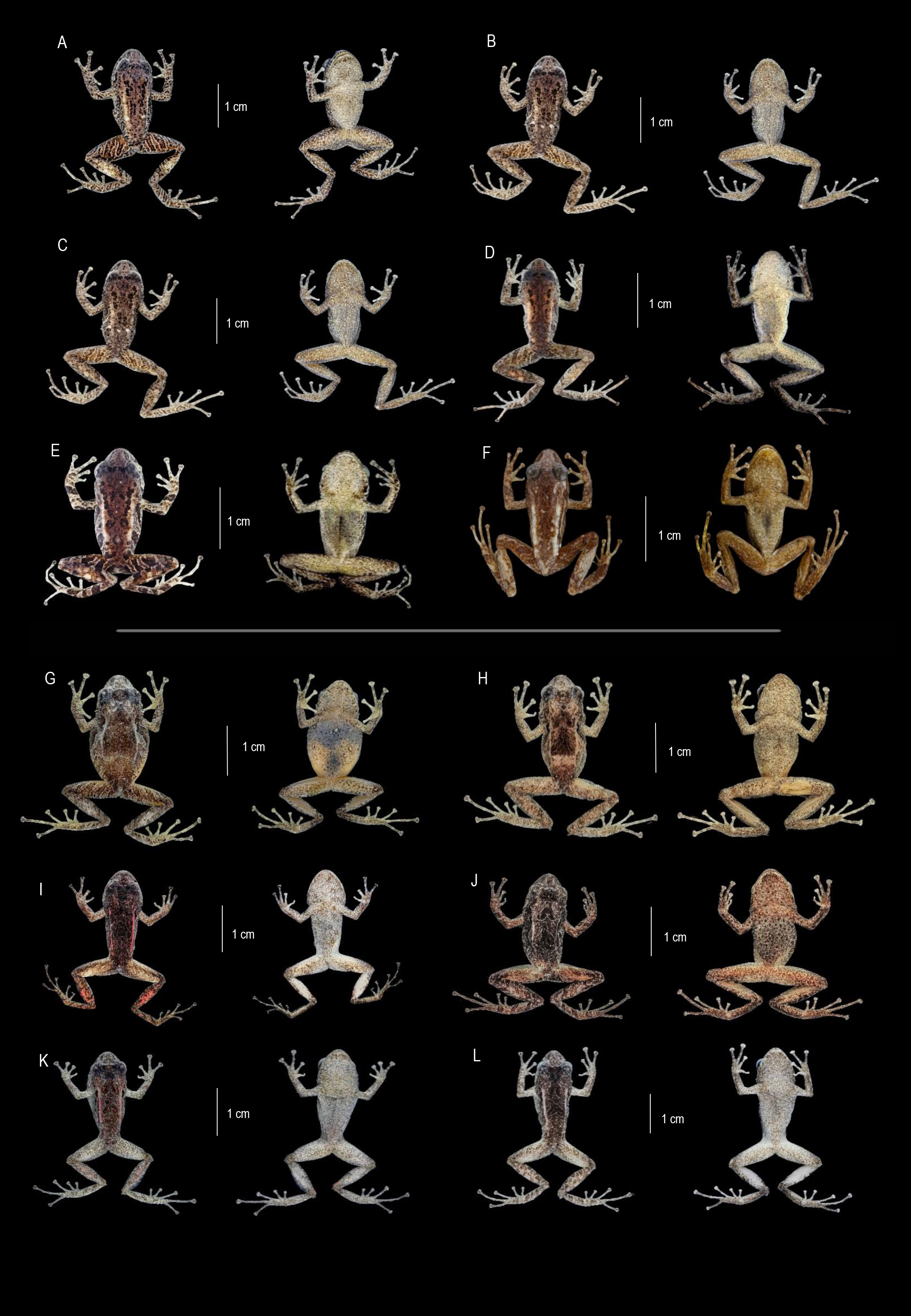

Supplement: Supplemental Information 8 — Holotype: (A) DHMECN 14858; Paratypes: (B) DHMECN 14885; (C) DHMECN 17903; (D) DHMECN 19538; (E) DHMECN 16578; (F) IAvH-Am-1492. Variation of Pristimantis mutabilis, (G) DHMECN 11755; (H) DHMECN 14796; (I) DHMECN 16433; (J) DHMECN 13279; (K) DHMECN 19479; (L) DHMECN 14978. Photographs by Christian Paucar. [file peerj-13-18680-s008.jpg]

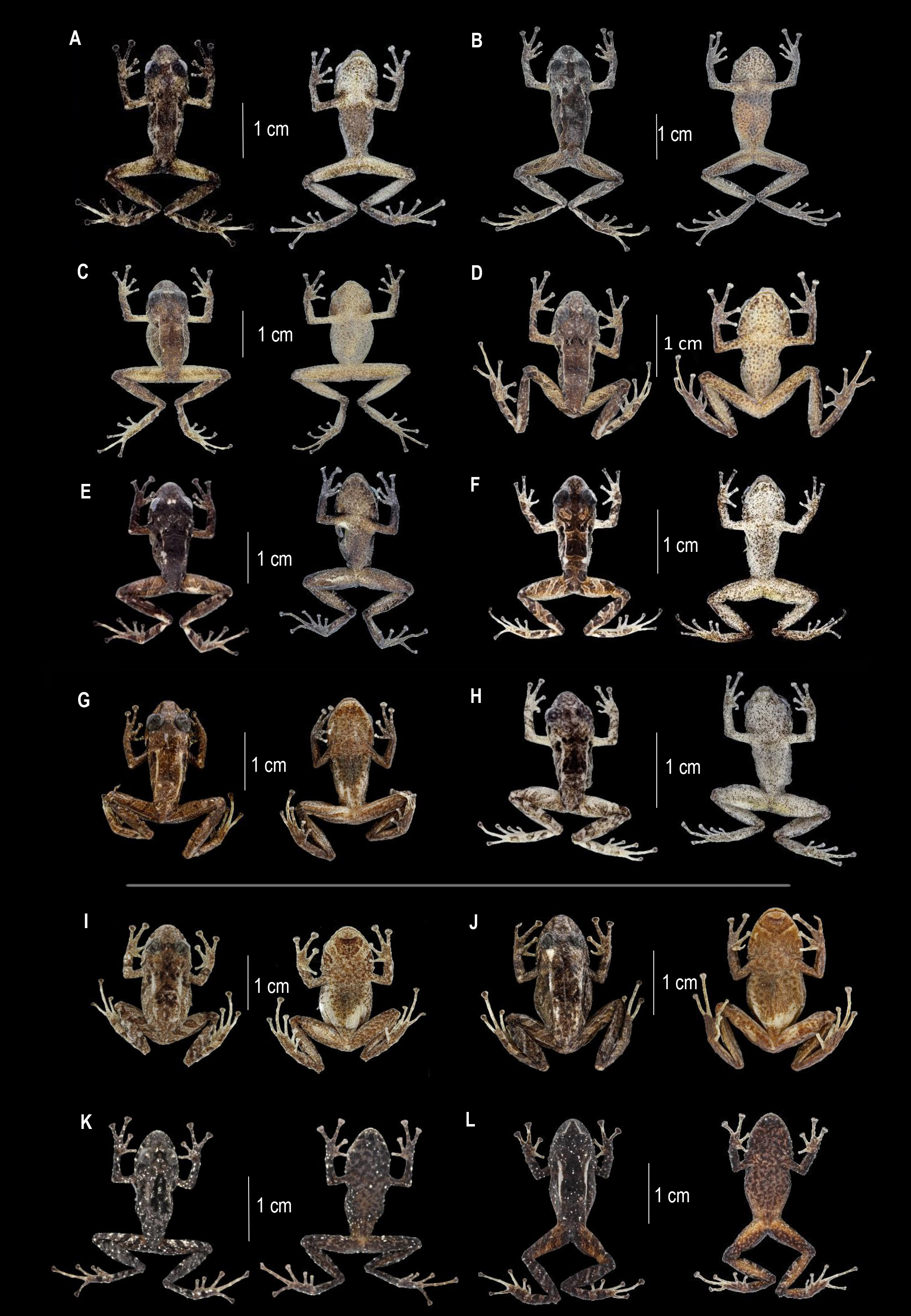

Supplement: Supplemental Information 9 — Holotype: (A) DHMECN 17894, Paratypes: (B) DHMECN 14960; (C) DHMECN 14979; (D) DHMECN 14886; (E) DHMECN 19429; (F) DHMECN 16567; (G) IAvH1801; (H) DHMECN 16575. Variation of type series of Eleutherodactylus verecundus. (I) IAvH1834, Paratypes: (J) IAvH1457; (K) DHMECN 19433; (L) DHMECN 5188). Photographs by Christian Paucar. [file peerj-13-18680-s009.jpg]

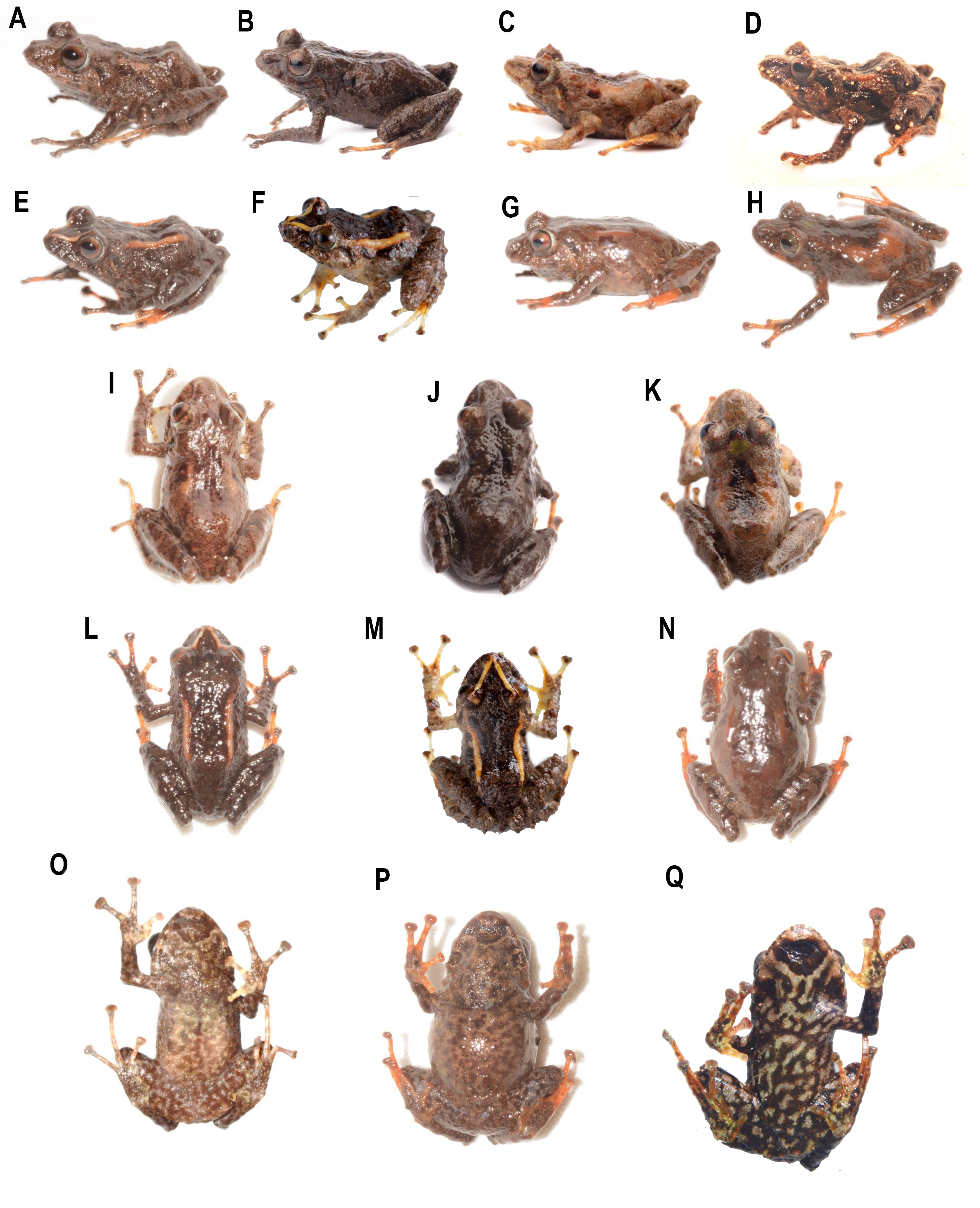

Supplement: Supplemental Information 10 — (A, I, O) DHMECN 12599, male, SVL: 18.77 mm; (B, J) DHMECN 17892, female, SVL:19.78 mm; (C, K,) DHMECN 17891, male, SVL: 18.85 mm; (D, Q) DHMECN 15007, male, SVL: 17.13 mm; (E, L) DHMECN 12500, male, SVL: 18.18 mm; (F, M) DHMECN 16569, female, SVL: 18.69; (G, N, P) DHMECN 12597; male, SVL: 18.50 mm; (H) DHMECN 12601, male, SVL: 16.43 mm. Photographs by Mario H. Yánez-Muñoz (A,D,E,G,H, I, L, N, O, P), Julio C. Carrión-Olmedo (B, C, J, K, Q), Jaime Culebras (F, M). [file peerj-13-18680-s010.jpg]
